# Human papillomavirus (HPV) vaccine coverage achievements in low and middle-income countries 2007–2016

**DOI:** 10.1016/j.pvr.2017.09.001

**Published:** 2017-10-03

**Authors:** Katherine E. Gallagher, Natasha Howard, Severin Kabakama, Sandra Mounier-Jack, Helen E.D. Burchett, D. Scott LaMontagne, Deborah Watson-Jones

**Affiliations:** a London School of Hygiene and Tropical Medicine, Clinical Research Department, Keppel St, London WC1E 7HT, United Kingdom; b Mwanza Intervention Trials Unit, National Institute for Medical Research, PO Box 11936, Mwanza, Tanzania; c London School of Hygiene and Tropical Medicine, Department of Global Health and Development, Keppel St, London WC1E 7HT, United Kingdom; d PATH, Center for Vaccine Innovation and Access, PO Box 900922, Seattle, WA 98109, United States

**Keywords:** HPV, Vaccine/vaccination, Coverage, Uptake, Completion, Low and middle income countries

## Abstract

**Introduction:**

Since 2007, HPV vaccine has been available to low and middle income countries (LAMIC) for small-scale ‘demonstration projects’, or national programmes. We analysed coverage achieved in HPV vaccine demonstration projects and national programmes that had completed at least 6 months of implementation between January 2007–2016.

**Methods:**

A mapping exercise identified 45 LAMICs with HPV vaccine delivery experience. Estimates of coverage and factors influencing coverage were obtained from 56 key informant interviews, a systematic published literature search of 5 databases that identified 61 relevant full texts and 188 solicited unpublished documents, including coverage surveys. Coverage achievements were analysed descriptively against country or project/programme characteristics. Heterogeneity in data, funder requirements, and project/programme design precluded multivariate analysis.

**Results:**

Estimates of uptake, schedule completion rates and/or final dose coverage were available from 41 of 45 LAMICs included in the study. Only 17 estimates from 13 countries were from coverage surveys, most were administrative data. Final dose coverage estimates were all over 50% with most between 70% and 90%, and showed no trend over time. The majority of delivery strategies included schools as a vaccination venue. In countries with school enrolment rates below 90%, inclusion of strategies to reach out-of-school girls contributed to obtaining high coverage compared to school-only strategies. There was no correlation between final dose coverage and estimated recurrent financial costs of delivery from cost analyses. Coverage achieved during joint delivery of HPV vaccine combined with another intervention was variable with little/no evaluation of the correlates of success.

**Conclusions:**

This is the most comprehensive descriptive analysis of HPV vaccine coverage in LAMICs to date. It is possible to deliver HPV vaccine with excellent coverage in LAMICs. Further good quality data are needed from health facility based delivery strategies and national programmes to aid policymakers to effectively and sustainably scale-up HPV vaccination.

## 1 Introduction

Persistent infection with high-risk human papillomavirus (HPV) genotypes is the cause of almost all cases of cervical cancer and is also associated with multiple other anogenital and oropharyngeal cancers [1]. Cervical cancer is the third most common cause of cancer-related deaths in women in low- and middle-income countries (LAMIC) [2]. In settings with effective screening programmes, most cervical abnormalities are identified and treated before they progress to cervical cancer. In many LAMIC the coverage of screening services is low [3] leading to women developing advanced stage disease and high cervical cancer mortality rates. Additionally, HIV, a major problem in many LAMIC, increases an individual's risk of HPV infection, persistence [4] and progression to cervical cancer [5], [6], [7], [8].

There are currently three prophylactic HPV vaccines. A bivalent vaccine targets HPV 16 and 18 (Cervarix®), and a quadrivalent vaccine (Gardasil®) additionally targets HPV 6 and 11 that cause genital warts. A nonavalent vaccine (Gardasil 9®) has recently been licensed in the US, Europe, and other high income countries and targets additional oncogenic HPV serotypes: 31, 33, 45, 52 and 58. All three vaccines have proven to be highly efficacious against persistent infection of their vaccine genotypes and associated cervical intraepithelial neoplasia [9], [10], [11]. The World Health Organisation (WHO) currently recommends vaccination of 9–13 year olds as vaccination is most effective prior to sexual debut and first exposure to HPV [12]. In 2014, the Strategic Advisory Group of Experts on Immunisation (SAGE) revised recommendations from a schedule of 3 doses [13], to 2 doses given at a 6–12 month interval [14] for girls aged 9–14 years [15], [16].

HPV vaccine first became available for use in LAMIC in 2007 with vaccine donations through the GARDASIL® Access Program (GAP) [17], manufacturer donations, the Bill & Melinda Gates Foundation through PATH, or other means. Demonstration projects were designed as pilot projects in small areas of a country to enable experience to be gained in delivering an expensive, gender-specific vaccine to, what was in many countries, a novel target age group [18]. In 2012, Gavi, the Vaccine Alliance, commenced support for HPV vaccination demonstration projects or national programmes in 53 Gavi-eligible countries [19]. Gavi support included some funds for formal evaluation of HPV vaccine delivery and required a coverage survey, post-introduction evaluation and costing analysis after the first year of implementation.

This paper presents the HPV vaccine coverage achieved in demonstration projects and national programmes in LAMIC that had completed at least six months of implementation between January 2007 and May 2016. Related publications summarising lessons learnt from HPV vaccination in 45 LAMICs have not had space to interrogate the substantial coverage data available [20], [21], [22].

## 2 Methods

### 2.1 Study design

This is a descriptive synthesis of data collected as part of a large study collating lessons learnt from HPV vaccine projects/programmes in LAMIC [20].

### 2.2 Country selection

A mapping exercise identified all low (LIC) and lower-middle income countries (LMIC) that were known to international organisations to have completed at least six months of a HPV vaccine demonstration or pilot project or an HPV vaccination national programme by May 2016, all were included in data collection. Data from upper-middle income countries (UMIC) that had completed demonstration project(s) in the same time period were also included. In total data were examined from 45 LAMIC for this study (Table 1; 18 LIC, 22 LMIC, 5 UMIC).

**Table 1:** Countries, projects, programmes approached during data collection.

**Country**	**Income Group**^a^	**Type of HPV vaccination financing**	**Country**	**Income Group**^a^	**Type of HPV vaccination financing**
**Bhutan**	LMIC	GAP demo and national programme (donation)	**Mali**	LIC	GAP and Gavi demos
**Bolivia**	LMIC	GAP demo	**Moldova**	LMIC	GAP demo
**Botswana**	UMIC	World Bank (WB), MOH demos and national programme (MOH)	**Mongolia**	LMIC	GAP demo
**Brazil**	UMIC	GAP, MOH demos and national (MOH)	**Mozambique**	LIC	Gavi demo
**Burkina Faso**	LIC	Gavi demo	**Nepal**	LIC	GAP/ACCF demo
**Cambodia**	UMIC	GAP demo	**Niger**	LIC	Gavi demo
**Cameroon**	LMIC	GAP and Gavi demos	**Papua New Guinea**	LMIC	GAP demo
**Cote d'Ivoire**	LMIC	Gavi demo	**Peru**	UMIC	PATH demo and national programme
**Ethiopia**	LIC	Gavi demo	**Philippines**	LMIC	Jhpiego demo
**The Gambia**	LIC	Gavi demo	**Rwanda**	LIC	National introduction (donation and Gavi)
**Georgia**	LMIC	GAP demo	**Senegal**	LMIC	Gavi demo
**Ghana**	LMIC	GAP and Gavi demos	**Sierra Leone**	LIC	Gavi demo
**Guyana**	LMIC	GAP demo and national programme	**Solomon Islands**	LMIC	Gavi demo
**Haiti**	LIC	GAP/PIH demo	**South Africa**	UMIC	demos and national programme
**Honduras**	LMIC	GAP demos and national programme (MOH)	**Tanzania**	LIC	GAP and Gavi demo
**India**	LMIC	PATH demo	**Thailand**	UMIC	Jhpiego demo
**Kenya**	LIC	GAP and Gavi demos	**Togo**	LIC	Gavi demo
**Kiribati**	LMIC	GAP/ACCF demo	**Uganda**	LIC	PATH, GAP, Merck demos and national programme (Gavi)
**Lao PDR**	LMIC	Gavi demo	**Uzbekistan**	LMIC	GAP demo
**Lesotho**	LMIC	GAP demo and national programme	**Vanuatu**	LMIC	ACCF demo and national programme
**Madagascar**	LIC	Gavi demo	**Vietnam**	LMIC	PATH demo
**Malawi**	LIC	Gavi demo	**Zambia**	LMIC	GAP demo
			**Zimbabwe**	LIC	Gavi demo

a World Bank classification February 2016. Abbreviations: ACCF, Australian Cervical Cancer Foundation; GAP, GARDASIL Access Programme; LIC, low-income country; LMIC, lower-middle-income country; MOH, Ministry of Health; PIH, Partners in Health; UMIC, upper-middle-income country.

### 2.3 Definitions

A ‘*demonstration project’* refers to a small-scale project, often limited to one or two districts or smaller administrative units in a country, and were defined by the funder and/or implementer and grant award details, e.g. GAP or Gavi or other funder. A ‘*programme*’ is a national vaccination programme. ‘*Delivery strategies*’ were defined by the vaccination sites used (schools, health facilities, outreach sites) and the target population (age or school grade). Within each demonstration project or national programme, if multiple different delivery strategies were piloted or the delivery strategy changed over time, these were defined as distinct *delivery experiences* (Supplementary Fig. 1). Countries often implemented multiple different projects/programmes over time and tested different delivery strategies so a country could have a number of different delivery experiences. More information on the different experiences is published elsewhere [20].

Uptake was defined as first dose coverage among the target population and was analysed alongside final dose coverage. Completion was defined as the proportion of girls who received the final dose of the vaccine schedule among those who had received the first dose. Coverage surveys were defined as surveys that used the WHO coverage survey guidelines [23] or similar, to assess the vaccination status of the general population.

### 2.4 Data collection

Methods are fully described elsewhere [20]. After obtaining informed consent, we conducted interviews over the phone or in person with key informants (KIs) e.g. EPI managers, HPV coordinators or equivalent, a systematic search of five databases for published literature and solicited unpublished documents including coverage surveys and country reports. Estimates of coverage results from projects/programmes were collated from written published and unpublished reports only. KIs and contacts supplying unpublished data were assured that the data would be anonymised to garner detailed reports on challenges as well as successes in implementing HPV vaccine delivery. Data were extracted onto a template informed by the WHO New Vaccine Introduction Guidelines [24]. National primary school enrolment data from the most recent year available were sourced from the UNESCO Institute of Statistics data centre [25].

### 2.5 Analysis

Coverage achievements were analysed descriptively by delivery experience. Due to the heterogeneity of funder requirements, project/programme organisation, design, and overall experience, multivariate analyses of the correlates of coverage were not appropriate. Where coverage and costing data were available, correlations were described. Reported coverage is for the selected target group of each distinct delivery experience. Where more than one estimate of coverage was available for the same delivery experience, the data considered to be of best quality were selected for descriptive analyses, e.g. estimates from coverage surveys were used wherever possible.

## 3 Results

A total of 56 KI interviews with representatives from 40 countries were completed, 188 unpublished documents were received, and 61 published articles and 11 conference abstracts identified. Lessons learnt on other topics have been published elsewhere [20], [21], [22], [26].

### 3.1 Data availability

**Fig. 1:**
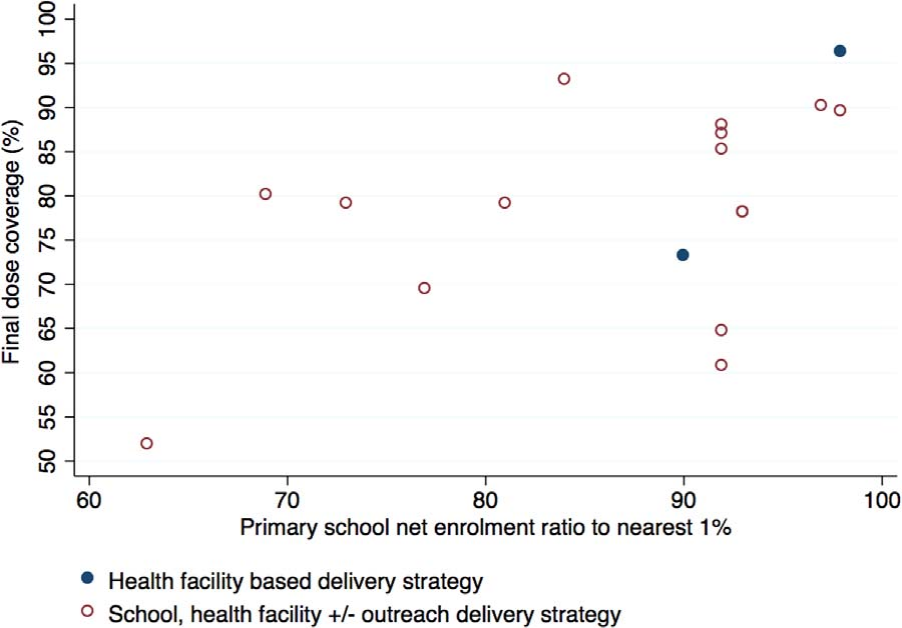
Coverage survey results (n = 17) from demonstration projects in 13 countries, plotted against estimates of primary school net enrolment ratios, by delivery strategy. Net primary school enrolment ratio: The number of children who belong to the age group that officially corresponds to that of primary schooling who are enrolled in primary school, divided by the total population of the same age group.

As of May 2016, only 17 delivery experiences in 13 countries had completed and finalised their coverage survey results (Fig. 1; Supplementary Fig. 2) [23]. All other coverage data came from administrative statistics that divided the reported number of doses administered by the estimated target population. Of the 91 distinct delivery experiences in 45 countries identified, 76 experiences (84%) across 40 LAMICs (89%) provided at least one estimate of uptake, completion, or final dose coverage. Over half (55%) of data were sourced from unpublished reports obtained from KI interviews for as there were no published coverage results available. Estimates for the remaining experiences were obtained from the published literature.

Estimates of uptake were available from 54 experiences in 35 countries and estimates of completion from 54 experiences in 30 countries. HPV vaccine final dose coverage estimates were available from 59 delivery experiences in 33 of the 45 countries [20].

### 3.2 Data accuracy

**Fig. 2:**
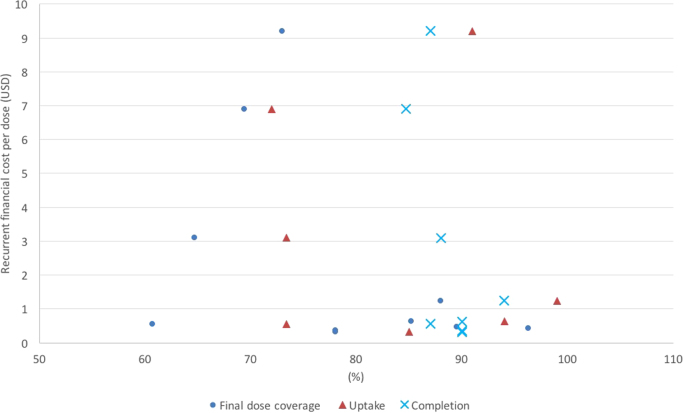
Uptake, coverage and completion achievements as documented in coverage surveys and the estimated recurrent financial cost of delivery per dose in 5 Gavi demonstration projects and 5 other demonstration projects. Recurrent financial cost of delivery per dose is presented as calculated in the source cost analyses. These analyses used different methods but were restricted to reporting costs likely to be ‘recurrent’ at every vaccination session i.e. not capital costs or start-up costs, and costs that were not already assumed by the routine immunisation system i.e. not economic costs; e.g. the additional allowances paid to staff for outreach activities specific to HPV vaccination were counted in the recurrent financial cost but core staff salaries were not included.

Across the 59 delivery experiences contributing final dose coverage data, the 42 administrative coverage estimates were, on average, higher than the 17 results from actual coverage surveys (Table 3). Only 8 countries reported both coverage survey results and administrative coverage data; 3 administrative coverage estimates were within 1% of the corresponding survey coverage Fig. 2 administrative coverage estimates were 6–7% lower than the subsequent coverage survey results, and 3 administrative coverage estimates were 5–17% higher than the subsequent survey data.

Reports and interviews indicated that administrative coverage data were of variable quality for a number of reasons. Census estimates were commonly used for the application to the vaccine provider in order to estimate the number of vaccine doses needed in the first year of the demonstration project/programme. However, during microplanning prior to delivery, challenges in estimating the true vaccine target population and therefore vaccine dose distribution to each district/health facility were experienced by almost all of the 41 countries that provided some information on uptake, coverage, or completion (Table 2). Additionally, where teachers, health-workers and/or community leaders did not fully understand the eligibility criteria, or could not practically operationalise it, doses were reportedly often administered to ineligible girls, e.g. vaccination of any girl over the age of 9 years when the target population was 9 year-olds only, or vaccination based on girls’ stature due to lack of age documentation. These challenges resulted in potential inaccurate estimations of both the number of doses administered to the target population and the size of the target population itself.

**Table 2:** Challenges in identifying and enumerating the HPV vaccine target population.

**Observation**	**Impact**
**Challenges in identifying and enumerating the HPV target population in schools**	
Incomplete Ministry of Education registration of all schools at district level.	Exclusion of new/private/religious or unregistered schools in microplanning and headcounts, resulting in a two-phase delivery of dose 1 as parents/teachers at unregistered schools in the area came forward later to request the vaccine.
Lack of adequate training/communication	Some schools included boys or ineligible girls in their headcounts.
Difficulty determining age	Stature or grade was used instead.
High rates of absenteeism	Inaccurate estimates of school grade populations led to more girls than expected on vaccination day.
**Challenges in identifying and enumerating the HPV target population out of school**	
Communities with out-of-school girls were generally hard to reach and difficult to identify.	Some countries used local social workers, community health workers or social enterprise/ NGO groups but house-to-house outreach was time intensive and expensive unless conducted by volunteers.
Intense social mobilisation encouraging any out of school girls to go to health facilities to be vaccinated yielded low coverage in this group.	Estimates of out of school girls were rarely verified/ validated.
Out of school girls are even less likely to know their age and eligibility.	Communication of eligibility criteria in non-interactive social mobilisation e.g. announcements and posters, needs to be adapted to reference commonly known events in the recent past to aid parents or guardians to determine age.

### 3.3 Coverage survey results

We obtained final dose coverage data from 17 coverage surveys in 13 countries; all were conducted after demonstration projects, 13 reported estimates of uptake and 12 reported data on completion. Nine coverage results from 5 countries were available in the published literature [27], [28], [29] and KIs provided 8 coverage estimates from 8 coverage surveys conducted after Gavi demonstration projects. Of the 17 survey results, two were from projects implementing health facility based delivery strategies [27], [28] and the remaining 15 were from projects that implemented ‘mixed strategies’ that predominantly used schools as vaccination venues alongside opportunities for vaccination outside schools, with or without specific additional community outreach (Fig. 1). Aside from one project, all were run with high levels of involvement from EPI and MOE officials. No coverage survey data were available from national HPV vaccine programmes.

The median final dose coverage across 17 coverage surveys was 79% (range 52–96%) the median uptake across 13 of the surveys was 88% (range 72–99%). It was possible to calculate 12 estimates of completion from the surveys with a median of 89% (range 71–94%).

Five of the surveys were after a 2-dose vaccination schedule and 12 were after a 3 dose schedule. There were no differences in the final dose coverage, uptake or completion rates achieved by vaccine schedule. The median coverage for the 2 dose schedule was 79% (range 65–93%) compared with 81.6% for the 3-dose schedules (range 52–96%). Only two estimates of completion were available for 2-dose schedules (87% and 88%); 10 were available for 3-dose schedules (median 90%, range 71–94%).

There were no correlations between survey data on final dose coverage and the estimated recurrent financial costs of delivery from cost analyses. Cost of delivery was estimated at US$1.11–2.10 per dose for 6 (non-Gavi supported) experiences in 2007–10, where coverage varied between 61% and 96% [27], [30]. The mean recurrent financial costs of delivery from 5 costing analyses performed on Gavi demonstration projects was US$6.05 per dose (range US$3.10–9.21); the corresponding coverage surveys for 4 of those countries indicated between 62% and 73% coverage (Fig. 2).

### 3.4 Administrative data

Administrative estimates of final dose coverage were available from 42 experiences in 20 countries (median 90%; range 51–105%). Forty-one delivery experiences in 22 countries provided administrative data on uptake with a median of 93% (not all of them also provided final dose coverage data; range 64–107%). Delivery experiences in 30 countries provided an estimate of completion, with/without coverage and uptake data, median 95% (range 70–100%; Table 3).

**Table 3:** All available final dose coverage, uptake and completion data by project/programme characteristic.

**Characteristic**	**Experiences with data**	**Number (%) of experiences with the following coverage:**					**Median (%; range)**
		**50–59%**	**60–69%**	**70–79%**	**80–89%**	≥ **90%**	
**Final dose coverage – administrative data**	42	2 (5)	4 (10)	3 (7)	11 (26)	22 (52)	90% (51–105)
**Final dose coverage – survey data**	17	1 (6)	3 (18)	5 (29)	5 (29)	3 (18)	79% (52–96)
**All final dose coverage data**^a^**:**							
** Country income group**^b^							
LIC	19	1 (5)	2 (11)	2 (11)	7 (37)	7 (37)	85% (51–105)
LMIC	28	1 (4)	4 (14)	4 (14)	5 (18)	14 (50)	90% (59–100)
UMIC	12	1 (8)	1 (8)	2 (17)	4 (33)	4 (33)	85% (51–98)
** Type of project/programme**							
National programme	8	0	1 (13)	0	1 (13)	6 (64)	92% (65–99)
Demonstration project	51	3 (6)	6 (12)	8 (16)	15 (29)	19 (37)	85% (51–105)
** Delivery strategy**							
School only	20	1 (5)	0	3 (15)	8 (40	8 (40)	86% (51–99)
School + health facility (± outreach)	34	2 (6)	5 (15)	4 (12)	8 (24)	15 (44)	87% (52–105)
Health facility only (± outreach)	5	0	2 (40)	1 (20)	0	2 (40)	73% (65–100)
** Joint delivery**							
Concurrent delivery^c^	6	0	2 (33)	0	0	4 (67)	91% (61–98)
None	46	3 (7)	3 (7)	8 (17)	15 (33)	17 (37)	85% (51–105)
** Dose schedule**							
2-dose	9	0	1 (12)	2 (22)	3 (33)	3 (33)	83% (65–98)
3-dose	50	3 (6)	6 (12)	6 (12)	13 (26)	22 (44)	88% (51–105)

		**Number (%) of experiences with the following uptake:**					**Median(%; range)**
		**50–59%**	**60–69%**	**70–79%**	**80–89%**	**>90%**	
**First dose uptake – administrative data**	41	0	1 (2)	3 (7)	12 (29)	25 (62)	93% (64–107)
**First dose uptake – survey data**	13	0	0	3 (23)	4 (31)	6 (46)	88% (72–99)
**All uptake data**^a^**:**							
** Country income group**^b^							
LIC	18	0	0	2 (11)	5 (28)	10 (56)	91% (70–100)
LMIC	25	0	0	3 (12)	8 (32)	14 (56)	94% (73–107)
UMIC	12	0	1 (8)	1 (8)	3 (25)	7 (58)	93% (64–101)
** Type of project/programme**							
National programme	8	0	0	1 (12)	2 (25)	5 (63)	92% (79–98)
Demonstration project	47	0	1 (2)	5 (11)	14 (30)	26 (55)	92% (70–107)
** Delivery strategy**							
School only	18	0	1 (6)	1 (6)	6 (33)	9 (50)	90% (70–100)
School + health facility (± outreach)	32	0	0	5 (16)	8 (25)	19 (59)	92% (72–107)
Health facility only (± outreach)	5	0	0	0	2 (40)	3 (60)	93% (82–101)
** Joint delivery**							
Concurrent delivery^c^	7	0	0	2 (29)	1 (14)	4 (57)	92% (72–99)
None	43	0	1 (2)	4 (9)	14 (32)	24 (55)	92% (70–107)
** Dose schedule**							
2-dose	12	0	1 (8)	4 (33)	7 (59)	0	91% (73–100)
3-dose	43	1 (2)	5 (12)	13 (30)	23 (54)	1 (2)	93% (70–107)

		**Number (%) of experiences with the following completion:**					**Median (%; range)**
		**50–59%**	**60–69%**	**70–79%**	**80–89%**	**>90%**	
**Completion – administrative data**	42	0	0	4 (9)	13 (31)	25 (60)	95% (70–100)
**Completion – survey data**	12	0	0	1 (8)	5 (42)	6 (50)	89% (71–94)
**All completion data**^a^**:**							
** Country income group**^b^							
LIC	17	0	0	3 (18)	6 (35)	8 (47)	88% (71–100)
LMIC	26	0	0	1 (4)	10 (38)	15 (58)	91% (70–100)
UMIC	11	0	0	1 (9)	2 (18)	8 (72)	94% (73–100)
** Type of project/ programme**							
National programme	6	0	0	0	1 (17)	5 (83)	97% (80–100)
Demonstration project	48	0	0	5 (10)	17 (35)	26 (54)	90% (70–100)
** Delivery strategy**							
School only	19	0	0	2 (11)	4 (21)	13 (68)	95% (75–100)
School + health facility (± outreach)	30	0	0	3 (10)	10 (33)	17 (57)	90% (70–100)
Health facility only (± outreach)	5	0	0	0	4 (80)	1 (20)	88% (80–100)
** Joint delivery**							
Concurrent delivery^c^	6	0	0	0	3 (50)	3 (50)	92% (85–100)
None	40	0	0	3 (7)	12 (30)	25 (63)	92% (70–100)
** Dose schedule**							
2-dose	4	0	0	0	2 (50)	2 (50)	92% (87–98)
3-dose	50	0	0	5 (10)	16 (32)	29 (58)	91% (70–100)

a If more than one coverage estimate was available from the same delivery experience the most reliable data were used e.g. coverage survey data were used when available.

b Excluded the HIC due to requirement for anonymity.

c This includes experiences that delivered another service at the same time as HPV vaccine (to the same age group). Row percentages.

Projects and programmes contributing administrative data were highly variable in organisation and evaluation requirements, e.g. some early GAP demos had little EPI or MOE involvement in project design and implementation and few formal evaluation requirements.

Administrative coverage data from 8 national programmes were available; 6 of these achieved ≥ 90% coverage (Table 3). Strategies involving schools as a vaccination venue predominated and achieved high coverage (Table 3).

Joint delivery of HPV vaccine alongside another intervention to the same age group was attempted in 14 delivery experiences. Only 6 supplied administrative data on coverage achievements; 4 experiences achieved > 90% coverage for HPV vaccination (Table 3). Interventions combined with HPV vaccination included those delivered in existing school health programmes or Child Health Days and re-introduction of interventions for this age group such as tetanus toxoid vaccination and/or deworming. There was little/no evaluation of the correlates of success of such strategies.

### 3.5 Correlates of uptake, completion and final dose coverage

As survey data were limited, we combined survey and administrative data to examine correlates of uptake, completion and coverage. All delivery experiences in 33 countries achieved more than 50% final dose coverage and almost half (42%) reported 90% or higher coverage with no trend over time (Table 3; Supplementary Fig. 2).

Even with combined data, only five estimates from health facility-based delivery strategies were available; the majority of experiences implemented school-based (n = 20, 33%) or mixed (n = 35, 58%) strategies. The predominance of data on delivery strategies using schools as a vaccination venue informed the analysis of correlates of good uptake, completion, and final dose coverage.

In 15 countries with ≥ 90% primary school net enrolment and coverage data, there was little difference in coverage achievements between those with or without an out-of-school strategy.

Among delivery experiences in countries with 80–89% primary school enrolment, those with an out-of-school strategy (10/22 experiences) gained a median final dose coverage of 95%. (inter-quartile range 90–98%) while those with no out-of-school strategy (12/22 experiences) had median coverage of 84% (interquartile range 80–92%). However, KIs reported challenges in identifying out-of-school girls. Specific mobilisation strategies for out-of-school girls were needed to make them aware of local opportunities to receive HPV vaccination. In addition to the involvement of teachers, where available, community health workers (CHWs) were reported to have provided a valuable resource in many different settings, in spreading educational messages about the vaccine, identifying out-of-school girls and following up girls who missed doses.

All four countries with less than 80% national primary school net enrolment and HPV vaccine demonstration project coverage data had implemented strategies to reach out-of-school girls. Some invited girls through tailored mobilisation strategies to schools and clinics for vaccination at the same time as school-going girls. KIs reported that outreach activities through fixed/mobile sites in the community and/or door-to-door vaccine delivery were critical to achieving any uptake in out-of-school girls. Community health workers (CHWs) aided mobilisation of communities prior to arrival of health workers for outreach sessions. Coverage in areas with low school enrolment was reportedly influenced by the difficulty of tracing vaccinated girls, with subsequent low completion rates.

Other factors reported in interviews to have influenced coverage included the social mobilisation strategy used, logistical challenges, and rumours (Table 4) [22]. Delays in vaccine delivery resulted in communities losing interest and were perceived to contribute to low coverage. Delays were prevented with high-level political commitment to ensure a smooth importation process, timely fund disbursement, and good inter-sectoral coordination.

**Table 4:** Factors correlated with high and low coverage experiences from KI interviews.

**HPV delivery experiences that achieved high/low final dose coverage**	**Factors correlated with coverage achieved, as reported in key informant interviews**
**‘High’ coverage (> 75%)**	• Strategies using schools that also had a good collaboration with the education sector at national and local levels (there are limited data on health facility only strategies which precludes correlation of factors to ensure their success). • Involvement of the national immunisation programme in planning and implementation. • Targeted social mobilisation of out-of-school girls to attend outreach venues for vaccination achieved uptake in this group, tracing out of school girls to ensure completion was challenging but successful in strategies that used community health workers and/or local volunteers. • Comprehensive social mobilisation of the whole community using face-to-face meetings with local ‘credible influencers’ (health workers, teachers, religious leaders, community elders). • Use of vaccination registers and cards aided tracing of girls to ensure completion. • Delivering vaccine on schedule (as communicated during social mobilisation) and within 1 school year (avoiding school exam time or vacation/harvest periods).
**‘Low’ coverage (50–75%)**	• Ineffective coordination and planning with schools, especially in areas of a high-proportion of private schools that generally needed more time and more intensive mobilisation than government schools. • Rumours that caused schools to refuse vaccinators. • Urban areas with high exposure to negative media/ mobile populations. • Other factors: Delay in receipt of social mobilisation and school-delivery funds, not providing a second opportunity for girls who missed the first dose.

KIs reported that achieving good coverage in urban areas was consistently more challenging than in rural areas as they were more exposed to negative media and rumours about the vaccine. Additionally, parents of girls attending private schools in general needed more information and longer to digest communication materials before accepting the vaccine compared to parents of girls attending government schools. The level and intensity of social mobilisation could vary across the country. In areas where rumours were underestimated, or not addressed immediately, these had a high impact on coverage attained and caused at least 3 projects to cease before they had completed delivering the third dose [22].

## 4 Discussion

This study built on findings in the published literature [27], [28], [29] by including substantial additional unpublished data, to form a comprehensive synthesis of HPV vaccine coverage achievements in LAMICs. Between 2007 and 2016, 33 of 45 LAMICs with HPV vaccination experience had final dose coverage data and all attained over 50% coverage in HPV vaccine demonstration projects or national programmes. These coverage achievements are aligned with, and in some cases exceed, coverage results from high income countries [31], [32]. However, there remains limited data from national programmes.

The majority of coverage data were administrative estimates; the advantages and disadvantages of using administrative data to monitor vaccine projects/programmes have been discussed in the published literature [33], [34]. However, we gained details around the specific challenges in the enumeration of the HPV vaccine target population.

Correlates of coverage were remarkably similar across country income groups, geographic regions and types of project/programme. In agreement with previously published literature, school-based delivery or a mixed strategy of school and health facility based delivery with/without outreach were the most common delivery strategies and obtained high coverage [35], [36]. There remains little experience and coverage data from strategies using only health facility-based delivery [27], [28], [36].

Findings from KI interviews confirmed and enriched the numerical data, providing additional insights into the multidimensional factors influencing uptake, completion and final dose coverage e.g. inter-sectoral collaboration, high-level political commitment and the implementation of different delivery strategies. The involvement of both CHWs and community leaders increased acceptance and uptake within the community as has been reported previously in Rwanda [37].

The comprehensive nature of this study allowed some previously published findings to be supported by data from many different contexts. It has been reported in at least 3 publications that urban communities may attain lower HPV vaccine coverage than rural or tribal areas [27], [38], [39].

Limitations include that 32 of the 92 delivery experiences that had completed at least 6 months of implementation by May 2016 in 12 countries were missing data on final dose coverage. Among those delivery experiences that reported coverage, 51 were demonstration projects, of relatively small scale and specifically designed to attain good coverage. The majority of data were administrative estimates with limited ability to assess data accuracy. Experiences that conducted coverage surveys may have differed in structure and organisation from those where only administrative data were available.

Coverage achievements did not seem to correlate with cost per dose delivered; further evaluation of how to optimise delivery strategies to achieve acceptable coverage at low cost is needed, as this is one of the key barriers to national introduction. Best practices when only delivering vaccine through health facilities and routine outreach services and/or during national programmes are not known. Concerns over the sustainability of the predominantly school-based delivery implemented in demonstration projects were widespread and are explored in related publications [20], [21], [26]. As countries transition away from Gavi financial support, policymakers have stated they need data and/or technical assistance to assess the relative cost and sustainability of different HPV vaccine delivery strategies in order for programmes to continue.

## 5 Conclusions

High HPV vaccine uptake, completion and final dose coverage has been achieved in demonstration projects and national programmes in 41 LAMICs to date. Data and interviews expose a multitude of factors that can influence uptake, completion and final dose coverage. Further good quality data are needed from health facility based delivery strategies and national programmes to aid policymakers to effectively and sustainably scale-up HPV vaccination.
