# Supplementary material for: Human papillomavirus (HPV) vaccine coverage achievements in low and middle-income countries 2007–2016
Source: Papillomavirus Res. 2017 Oct 3;4:72–8. doi: 10.1016/j.pvr.2017.09.001 (PMC5710977; doi:10.1016/j.pvr.2017.09.001)

Supplementary Fig. 1: The number of countries, programmes/projects and delivery experiences included in the study

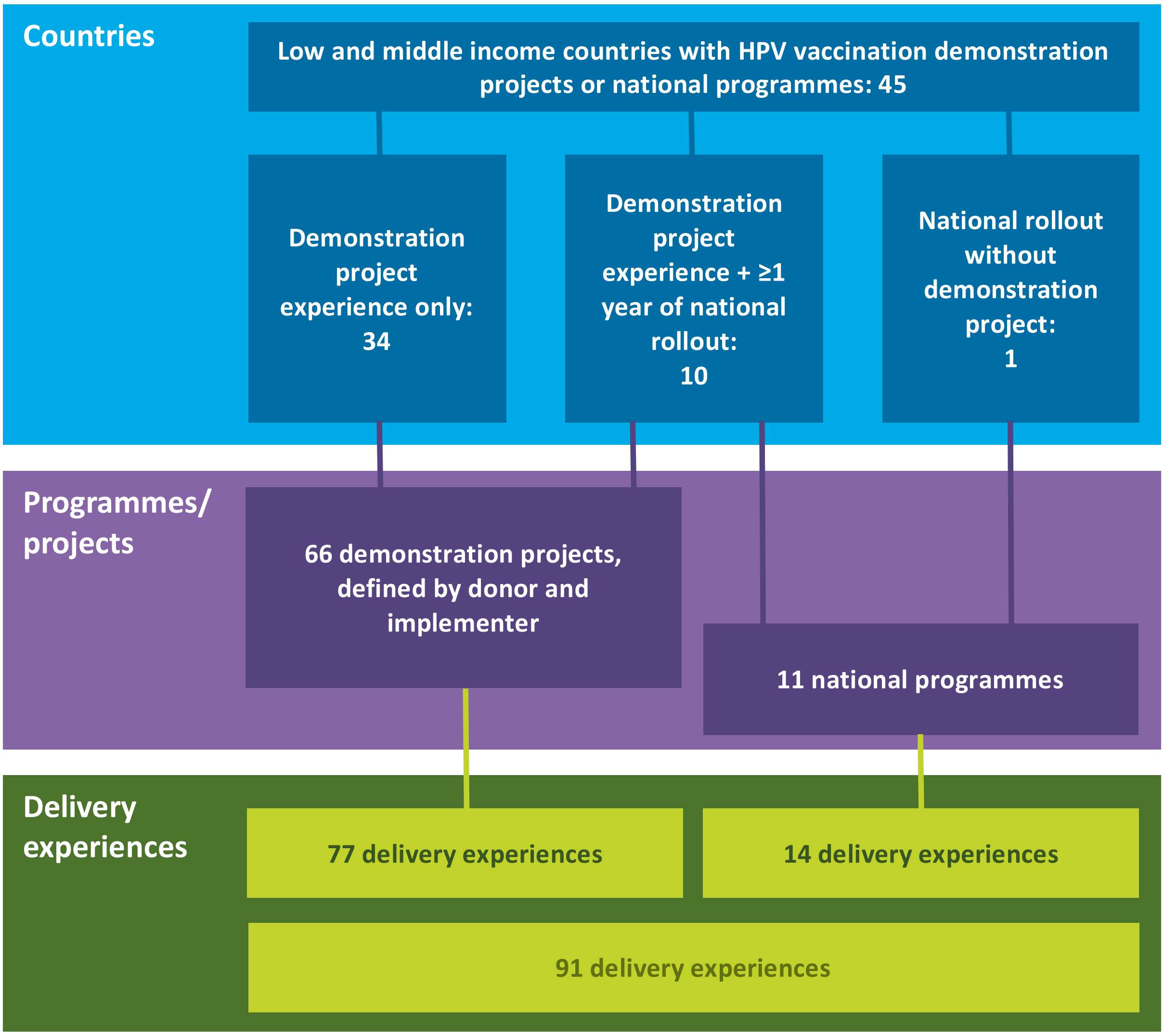

Supplement: Supplementary file 1 — Supplementary material [file mmc1.pdf]
