# Supplementary material for: Human papillomavirus (HPV) vaccine coverage achievements in low and middle-income countries 2007–2016
Source: Papillomavirus Res. 2017 Oct 3;4:72–8. doi: 10.1016/j.pvr.2017.09.001 (PMC5710977; doi:10.1016/j.pvr.2017.09.001)
Supplement: Supplementary file 2 — Supplementary material [file mmc2.pdf]

Supplementary Figure 2. All available final dose coverage estimates from surveys and administrative data, national programmes and demonstration projects across time[1]

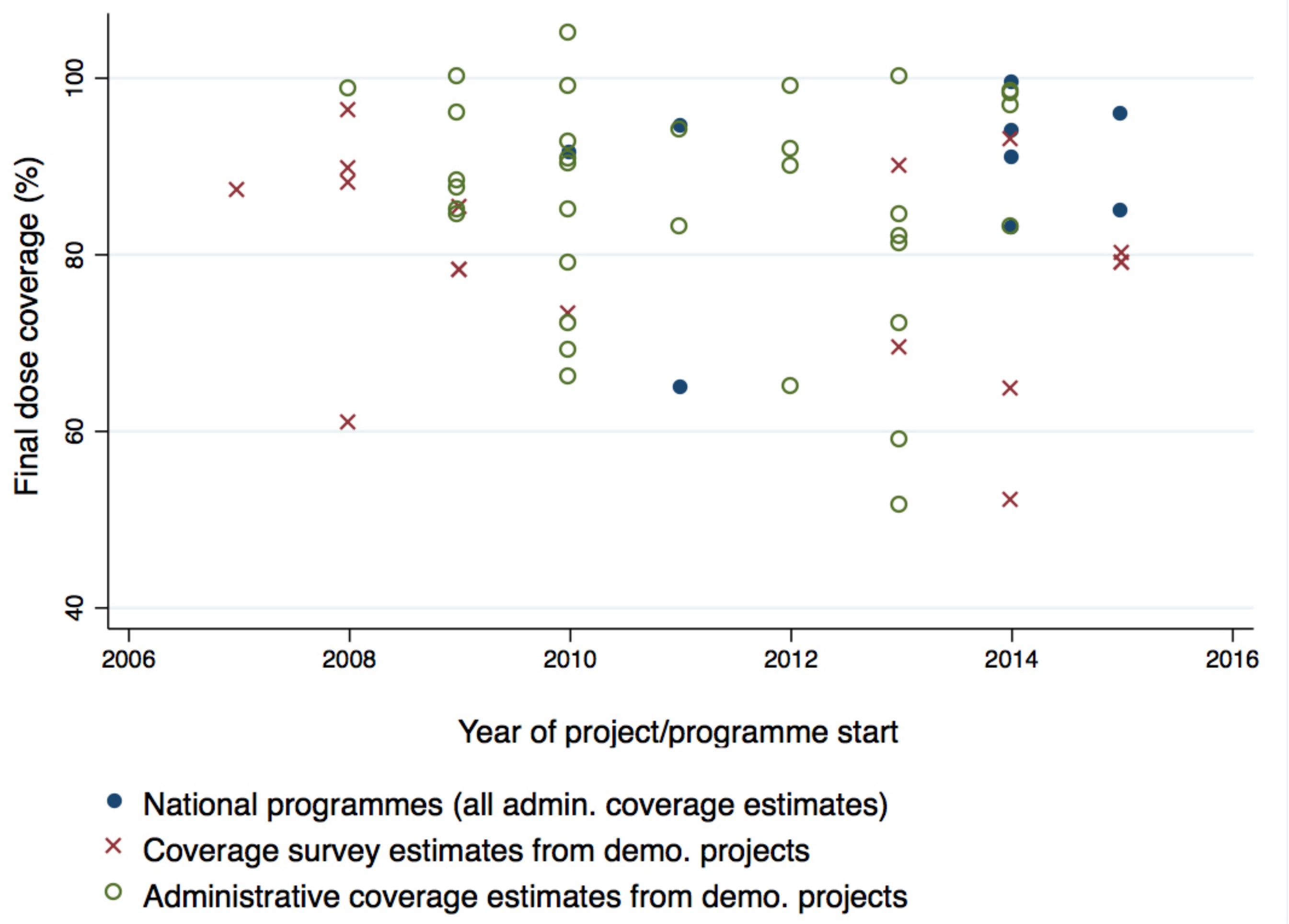

[1]Where more than one estimate of coverage was available for a single delivery experience, survey data, or the most reliable data available, were used.
